# Binary Particle Swarm Optimization with Manta Ray Foraging Learning Strategies for High-Dimensional Feature Selection

**DOI:** 10.3390/biomimetics10050315

**Published:** 2025-05-13

**Authors:** Jianhua Liu, Yuxiang Chen, Shanglong Li

**Affiliations:** 1 School of Artificial Intelligence, Xiamen Institute of Technology, Xiamen 361021, China; 2 School of Computer Science and Mathematics, Fujian University of Technology, Fuzhou 350118, China; 2211308006@smail.fjut.edu.cn (Y.C.); 2231308056@smail.fjut.edu.cn (S.L.)

**Keywords:** feature selection, high-dimensional data, binary particle swarm optimization, manta ray foraging optimization, search phases

## Abstract

High-dimensional feature selection is one of the key problems of big data analysis. The binary particle swarm optimization (BPSO) method, when used to achieve feature selection for high-dimensional data problems, can get stuck in local optima, leading to reduced search efficiency and inferior feature selection results. This paper proposes a novel BPSO method with manta ray foraging learning strategies (BPSO-MRFL) to address the challenges of high-dimensional feature selection tasks. The BPSO-MRFL algorithm draws inspiration from the manta ray foraging optimization (MRFO) algorithm and incorporates several distinctive search strategies to enhance its efficiency and effectiveness. These search strategies include chain learning, cyclone learning, and somersault learning. Chain learning allows particles to learn from each other and share information more effectively in order to improve the social learning ability of the population. Cyclone learning introduces a gradual increase over iterations, which helps the BPSO-MRFL algorithm to transition smoothly from exploratory searching to exploitative searching, and it creates a balance between exploration and exploitation. Somersault learning enables particles to adaptively search within a changing search range and allows the algorithm to fine-tune the selected features, which enhances the algorithm’s local search ability and improves the quality of the selected subset. The proposed BPSO-MRFL algorithm was evaluated using 10 high-dimensional small-sample gene expression datasets. The results demonstrate that the proposed BPSO-MRFL algorithm achieves enhanced classification accuracy and feature reduction compared to traditional feature selection methods. Additionally, it exhibits competitive performance compared to other advanced feature selection methods. The BPSO-MRFL algorithm presents a promising approach to feature selection in high-dimensional data mining tasks.

## 1. Introduction

High-dimensional data denote datasets characterized by a large number of features or attributes, wherein the dimensionality typically exceeds the number of observations. However, such data present significant challenges, including the “curse of dimensionality”. This phenomenon adversely impacts the efficacy of conventional data analysis and modeling methodologies, as the distances between data points become increasingly diffuse and sparse in high-dimensional spaces. Consequently, this results in heightened susceptibility to overfitting and elevated computational expenses [1]. In the analysis of high-dimensional data, feature selection is an essential step to reducing model complexity and computational costs and enhance the model’s generalization capability. By eliminating redundant and irrelevant features, feature selection minimizes noise interference, thereby improving the accuracy and robustness of predictive models. Furthermore, it facilitates a deeper understanding of the underlying data structure, enabling the identification of latent patterns and relationships within high-dimensional datasets. As such, feature selection constitutes a critical component in the preprocessing and modeling of high-dimensional data, contributing significantly to both interpretability and performance [2].

In fact, feature selection (FS) is a combinatorial optimization problem that aims to optimize two conflicting objectives: maximizing the accuracy of feature classification and minimizing the number of selected features [3]. Dealing with high-dimensional data remains a challenging task due to the fact that the search space grows exponentially with the number of features. Feature selection methods can broadly be classified into three categories: wrapper methods [4,5], filter methods [6], and embedded methods [7,8]. Filter methods evaluate the importance of features regardless of any specific learning algorithm, making them computationally efficient and capable of handling a large number of features. However, they may not capture dependencies between features or their interaction with the learning algorithm. Filter methods are also prone to overfitting, and the selected features may not necessarily lead to the best performance in a specific learning task. Wrapper methods use a specific learning algorithm to evaluate the usefulness of features. They select a subset of features by repeatedly training and testing the learning algorithm with different subsets of features. Wrapper methods are computationally expensive and may overfit the data, but they are often more effective than filter methods in selecting relevant features for a specific learning task. Embedded methods incorporate feature selection into the learning algorithm itself. These methods typically use regularization techniques, such as Lasso or Ridge regression, to penalize the coefficients of irrelevant features and encourage sparsity in the resulting model. Embedded methods are computationally efficient and can produce models with good predictive performance. However, they may require a large amount of data and may not work well with non-linear models.

The wrapper-based approach to feature subset selection constitutes an NP-hard problem. Although exhaustive search strategies that evaluate all possible subsets can theoretically identify the optimal solution, their computational complexity renders them impractical for most real-world applications. Consequently, efficient global search techniques are necessary to navigate the expansive solution space effectively. Meta-heuristic algorithms have emerged as particularly suitable methods for resolving such complex combinatorial optimization challenges. Empirical studies have demonstrated the efficacy of swarm intelligence algorithms—a prominent subclass of meta-heuristics—in addressing feature selection problems [9]. Notable swarm-intelligence-based optimization techniques applied to this domain include gray wolf optimization (GWO) [10], particle swarm optimization (PSO) [11], gravitational search algorithms (GSAs) [12], and genetic algorithms (GAs) [13], each offering distinct advantages in feature subset exploration and selection.

In recent years, binary particle swarm optimization (BPSO) has gained significant attention due to its conceptual simplicity, ease of implementation, and fast convergence. It has also been successfully applied to solve feature selection problems [14]. BPSO was proposed by Kennedy and Eberhart in 1997 [15]. PSO was converted into BPSO by using a transfer function that maps a continuous search space to a binary one. The updating process is designed to switch the positions of particles between 0 and 1 in binary search spaces. The goal of feature selection is to eliminate as many irrelevant and redundant features as possible. To achieve this goal, existing wrapper-based feature selection methods based on BPSO typically adopt an integrated fitness function that combines maximizing classification performance and minimizing feature subset size [16]. However, with an increase in data dimension, the search space of the feature selection problem grows exponentially. This leads to a problem that satisfactory feature subsets with some key features may not be found, as a large number of feasible feature subsets pose significant challenges to BPSO. BPSO algorithms often search too slowly to obtain good feature subsets. So, further improvements to the exploration and exploitation capabilities of the BPSO algorithm are needed.

Researchers have proposed various BPSO algorithms that use different mechanisms to enhance the search process. Mirjalili et al. [17] introduced six new transfer functions categorized into two types, S-shaped and V-shaped, and significantly enhanced the original BPSO algorithm. Xue et al. [18] proposed a variable-length representation method for PSO-based feature selection. Although these strategies made PSO obtain better solutions in less time, the computational cost of this method was high. Song et al. [19] proposed the Mutual Information-based Bare-Bones PSO algorithm (MIBBPSO). This algorithm employed an effective population initialization strategy based on label correlation, which utilized the correlation between features and labels to accelerate population convergence. However, when solving high-dimensional problems, this method required a significant amount of time, and due to the limitations of the initialization strategy, the diversity of particles was constrained, leading to a higher likelihood of the algorithm getting trapped in local optima. Jain et al. [20] proposed a hybrid method by combining the Correlation-based Feature Selection (CFS) technique with an improved BPSO algorithm. This method also faces the same problem as MIBBPSO. Thaer et al. [21] proposed a method called Boolean particle swarm optimization with evolutionary population dynamics. Six natural selection mechanisms, including the Best-Based, Tournament, Roulette Wheel, Stochastic Universal Sampling, Linear Rank, and Random-Based mechanisms, were employed to select better solutions. Because of the adoption of multiple selection mechanisms, this algorithm also had high computational costs. Cheng et al. [22] introduced competitive particle swarm optimization (CSO). CSO enabled particles to learn from better particles randomly selected from the swarm, leading to improved performance. Due to the introduction of a competitive mechanism, the algorithm still incurs significant computational costs when dealing with high-dimensional problems. In summary, the recently proposed algorithms have all improved the performance of BPSO in feature selection. However, their ability to handle high-dimensional datasets is still limited. Therefore, further research is necessary.

In addition to BPSO, researchers have also investigated the application of other non-PSO-based metaheuristic algorithms in feature selection. Genetic algorithms (GAs) have emerged as one of the most popular metaheuristic algorithms used to solve FS problems. Oh et al. [23] proposed a GA-based FS method that incorporated local search operations and genetic operations, but this method was prone to premature convergence and exhibited low search efficiency. Emary et al. [24] introduced a feature selection method based on the gray wolf optimization (GWO) algorithm, but this algorithm had poor population diversity and exhibited slow convergence speed in later stages. RYM Nakamura et al. [25] proposed a binary bat algorithm for feature selection, which combined the exploration ability of the bat algorithm with the Best Path Forest classifier. Majdi et al. [26] proposed two binary variants of the Whale Optimization Algorithm (WOA). The first variant explored the impact of using Tournament and Roulette Wheel selection mechanisms during the search process, and the second variant incorporated crossover and mutation operators to enhance the exploitation ability of the WOA algorithm. Hichem et al. [27] introduced a novel binary variant of the Grasshopper Optimization Algorithm (GOA). Their algorithm initialized the positions of grasshoppers with binary values and employed simple operators for position updates. Ibrahim et al. [28] introduced eight time-varying S-shaped and V-shaped transfer functions into the Binary Dragonfly Algorithm (BDA). These metaheuristic algorithms mentioned above also face similar challenges as the variants of binary particle swarm optimization algorithm. They have high computational costs, the convergence speed is slow, and it is easy to get trapped in local optima when dealing with high-dimensional problems.

Furthermore, numerous hybrid metaheuristic algorithms have been proposed to deal with feature selection problem. For instance, Qasem et al. [29] presented a binary gray wolf optimization–particle swarm optimization hybrid algorithm, which combining PSO and GWO. Ranya et al. [30] proposed a binary hybridization of GWO and Harris hawks optimization. This method employed an S-shaped transfer function to convert the continuous search space into a binary search space. Zohre et al. [31] introduced a three-stage hybrid feature selection method called information gain-based butterfly optimization algorithm (BOA). This method combined information gain technique with the BOA. Lu et al. [32] proposed a hybrid feature selection technique that integrated mutual information maximization and adaptive genetic algorithm. Ma et al. [33] proposed a two-stage hybrid ant colony algorithm. This algorithm incorporated an interval strategy to determine the optimal subset size of features searched in the additional stage. Overall, hybrid metaheuristic algorithms are also efficient and effective in finding the best subset of features for classification. However, when applied to high-dimensional feature selection problems, these algorithms still suffer from high computational overhead and low convergence accuracy. Thus, there is still room for further improvement in the convergence speed and accuracy of these algorithms.

Manta ray foraging optimization (MRFO) [34] is a swarm intelligence algorithm inspired by the foraging behaviors of manta rays. Specifically, the optimization process of MRFO involves three foraging operators: chain foraging, cyclone foraging, and somersault foraging. The chain foraging strategy makes each individual update its position with respect to the one in front of it and the current global best solution. The cyclone foraging strategy makes each individual update its position with respect to both the one in front of it and a reference position, which can either the best position obtained so far or a random position produced in the search space. The choice between the two depends on the value of iteration. The gradual increase in the value of iteration encourages MRFO to smoothly transition from an exploratory search to an exploitative search. With the value of the random number, MRFO can switch between chain foraging and cyclone foraging. Somersault foraging allows individuals to adaptively search in a changing search range.

The MRFO has been widely applied. Abdel-Mawgoud et al. [35] proposed an improved manta ray foraging optimizer, introducing a simulated annealing operator to enhance the development phase of MRFO and applying it to solve the integration of renewable energy in distribution networks. Supiksha et al. [36] combined the MRFO with the rider optimization algorithm, proposing general adversarial networks based on a manta ray foraging optimizer for effective glaucoma detection. Ibrahim et al. [37] proposed a hybrid improved bat foraging optimization algorithm with the slap swarm algorithm to address IoT task scheduling issues in cloud computing. Yuxian et al. [38] proposed an enhanced elephant herding optimization algorithm and introduced the rolling foraging strategy of bats and Gaussian mutation. Neeraj et al. [39] utilized the MRFO to optimize multiple locally relevant embedding strength values (MESs), balancing invisibility and robustness, and proposed a novel image adaptive watermarking scheme called MantaRayWmark.

In this paper, we propose a novel algorithm called the BPSO-MRFL, which integrates the MRFO search mechanism into BPSO. BPSO-MRFL involves three search phases—chain learning, cyclone learning, and somersault learning—which enhances the algorithm’s search ability and obtains better performance.

The novelty of this work is as follows:

1. The algorithm introduces the chain learning of the manta ray foraging optimization algorithm into the binary particle swarm optimization algorithm, which effectively improves the exploration ability of the algorithm;
2. The algorithm introduces the rolling learning of the manta ray foraging optimization algorithm into the binary particle swarm optimization algorithm, which effectively improves the development ability of the algorithm;
3. The algorithm introduces the whirlwind learning of the manta ray foraging optimization algorithm into the binary particle swarm optimization algorithm to balance the development and exploration ability of the algorithm;
4. We conduct experiments on 10 gene expression datasets with thousands of features that are publicly available to evaluate the performance of the proposed algorithms;

The remainder of this paper is organized as follows. Section 2 presents the background and related work. Section 3 introduces the proposing BPSO-MRFL algorithm. Section 4 presents the experimental datasets, the evaluating metrics of experimental results, and the parameter settings of the comparing algorithms. Section 5 provides the experimental results and comparisons. Finally, Section 6 presents the conclusions of this paper.

## 2. Related Work

### 2.1. Binary Particle Swarm Optimization

PSO is a swarm intelligence algorithm inspired by the flocking behavior of birds in nature. In the PSO algorithm, each particle is characterized by a position vector and a velocity vector, and it explores the solution space based on its current best-known position. Additionally, particles share information regarding the best positions discovered by the entire swarm. The velocity and position of each particle are updated according to Equations (1) and (2).

(1) $$v_{i}^{t+1}=wv_{i}^{t}+c_{1}\times r_{1}\times \left({pbest}_{i}-x_{i}^{t}\right)+c_{2}\times r_{2}\times \left(gbest-x_{i}^{t}\right)$$

(2) $$x_{i}^{t+1}=x_{i}^{t}+v_{i}^{t+1}$$

where $v_{i}^{t}$ and $v_{i}^{t+1}$ denote the velocity of the *i*-th particle at the *t*-th and the (*t* + 1)-th generations, respectively; *w* is the inertia weight; *c*_1_ and *c*_2_ are two learning factors that control the particles’ learning rates; *pbest_i_* represents the personal best position of the *i*-th particle; *gbest* is the global best position found by the swarm; *r*_1_ and *r*_2_ are two random numbers uniformly distributed in the interval [0, 1]; and $x_{i}^{t}$ and $x_{i}^{t+1}$ represent the position of the *i*-th particle at the *t*-th and (*t* + 1)-th generations, respectively.

A sigmoid function, defined in Equation (3), is introduced to map real values into the interval [0, 1]. This function, referred to as the transfer function, plays a crucial role in BPSO.

(3) $$Tf=\frac{1}{1+\exp\left({-v}_{i}^{t+1}\right)}$$

The transfer function defined in Equation (3) generates a value within the range [0, 1], and the particle’s position in the binary search space is determined based on Equation (4).

(4) $$x_{i}^{t+1}=\left\{\begin{array}{c} 1\;\;\;\;\;\mathrm{If}\;rand<\mathrm{Tf}\\ 0\;\;\;\;\;\mathrm{If}\;rand\geq \mathrm{Tf} \end{array}\right.$$

In PSO, the velocity represents the proximity of the current position to the global optimum. A small velocity indicates that the particle is close to the global solution, and thus, its next position should be updated with a minor adjustment. Conversely, a large velocity suggests that the particle requires larger movements. Unlike PSO, BPSO does not update a particle’s position based on its current position. Instead, in BPSO, a positive velocity increases the probability that a bit in the particle’s position will be one, while a negative velocity increases the likelihood that the bit will be zero. Additionally, when the velocity is zero, the value of the sigmoid function is 0.5, leading to an equal probability of the bit being zero or one. As a result, BPSO may exhibit divergence toward the end of the algorithm, since the particle position bits are determined independently of their previous states.

### 2.2. Manta Ray Foraging Optimization

Manta ray foraging optimization (MRFO) is inspired by the intelligent foraging behaviors of manta rays. To simulate these behaviors, the algorithm incorporates three distinct foraging strategies: chain foraging, cyclone foraging, and somersault foraging.

In chain foraging, individuals form a chain and move toward areas with higher plankton concentration, while also following the individual directly ahead of them. The mathematical modeling of chain foraging is described by Equations (5) and (6).

(5) $$q_{i}^{t+1}=\left\{\begin{array}{c} q_{i}^{t}+r_{1}\left(q_{b}-q_{i}^{t}\right)+\alpha \left(q_{b}-q_{i}^{t}\right)\;\;\;\;\;\;\;\;\;\;\;\;\;\;i=1\\ q_{i}^{t}+r_{2}\left(q_{i-1}^{t}-q_{i}^{t}\right)+\alpha \left(q_{b}-q_{i}^{t}\right)\;\;\;\;\;\;\;\;i=2,\ldots ,N \end{array}\right.$$

(6) $$\alpha =2r_{3}\sqrt{\left|\log{(r}_{4})\right|}$$

where $q_{i}^{t}$ is the current position of the *i*-th manta ray in the *t*-th generation; *q_b_* is the global best position of all manta rays; and α is a weight coefficient.

In cyclone foraging, manta rays in deep water form a spiral-shaped foraging chain toward a patch of plankton. In addition to spiraling toward the food source, each manta ray also moves toward the individual ahead of it. The mathematical modeling of the spiral movement of manta rays in a two-dimensional space is described by Equations (7) and (8).

(7) $$q_{i}^{t+1}=\left\{\begin{array}{c} q_{b}+r_{5}\left(q_{b}-q_{i}^{t}\right)+\beta \left(q_{b}-q_{i}^{t}\right)\;\;\;\;\;\;\;\;\;\;\;\;\;i=1\\ q_{b}+r_{6}\left(q_{i-1}^{t}-q_{i}^{t}\right)+\beta \left(q_{b}-q_{i}^{t}\right)\;\;\;\;\;\;i=2,\ldots ,N \end{array}\right.$$

(8) $$\beta =2e^{r_{8}}\times \left(\frac{T-t+1}{t}\right)\sin\left(2\pi \times r_{8}\right)$$

where β is a weight coefficient and *T* is the maximum number of iterations.

The MRFO algorithm employs cyclone foraging behavior to balance exploitation and exploration during the search process. Individuals move along a spiral path toward both the food source and the individual ahead of them. To enhance exploration capability, individuals are occasionally assigned a new random reference position within the search space. This mechanism enables MRFO to perform an extensive global search. The corresponding mathematical modeling is described by Equations (9) and (10).

(9) $$q_{rp}^{t}=Lw+r_{9}\left(Up-Lw\right)$$

(10) $$q_{i}^{t+1}=\left\{\begin{array}{c} q_{rp}^{t}+r_{10}\left(q_{rp}^{t}-q_{i}^{t}\right)+\beta \left(q_{rp}^{t}-q_{i}^{t}\right)\;\;\;\;\;\;\;\;\;i=1\\ q_{rp}^{t}+r_{11}\left(q_{i-1}^{t}-q_{i}^{t}\right)+\beta \left(q_{rp}^{t}-q_{i}^{t}\right)\;\;\;i=2,\ldots ,N \end{array}\right.$$

where $q_{rp}^{t}$ is a randomly generated position within the search space range and *Lw* and *Up*, respectively, represent the lower and upper bounds of the search space.

In somersault foraging, manta rays treat the food’s position as a pivot point, swimming around it and updating their positions relative to the best solution found so far. The corresponding mathematical model is given in Equation (11).

(11) $$q_{i}^{t+1}=q_{i}^{t}+S\times \left(r_{12}\times q_{b}-r_{13}\times q_{i}^{t}\right)$$

where *S* is the somersault factor and its value is set to 2.

## 3. Binary Particle Swarm Optimization with Manta Ray Foraging Learning Strategies (BPSO-MRFL) Algorithm

This section proposes the binary particle swarm optimization with manta ray foraging learning strategies (BPSO-MRFL) for feature selection. The proposed algorithm aims to enhance the performance of the BPSO algorithm in selecting features for high-dimensional datasets. Three different learning strategies of the MRFO algorithm are introduced into the BPSO algorithm, which include chain learning, cyclone learning, and somersault learning. The following subsections will provide a detailed explanation on each learning strategy.

### 3.1. Chain Learning

In the chain learning phase, the particles work like the manta rays of MRFO. They line up head-to-tail and form a foraging chain. The particles, except for the first one, move towards not only the best particle but also the one in front of them. The velocity update formula is shown in Equation (12).

(12) $$v_{i}^{t+1}=\left\{\begin{array}{c} \omega v_{i}^{t}+c_{1}{\times r}_{1}\times \left({pbest}_{i}-x_{i}^{t}\right)+c_{2}{\times r}_{2}\times \left(gbest-x_{i}^{t}\right)\;\;\;\;\;\;\;i=1\\ \omega v_{i}^{t}+c_{1}\times r_{1}\times \left({pbest}_{i-1}-x_{i}^{t}\right)+c_{2}\times r_{2}\times \left(gbest-x_{i}^{t}\right)\;\;i=2,\ldots ,N \end{array}\right.$$

where $v_{i}^{t}$ is the velocity of the *i*-th particle in the previous iteration; *pbest_i_* and *gbest* is the personal best position and global best position of the *i*-th particle; *r*_1_ and *r*_2_ are two different random numbers in the interval [0, 1]; *c*_1_ and *c*_2_ are learning factors; and *w* is a linearly decreasing inertia weight. The updated formulas of *c*_1_ and *c*_2_ are shown in Equations (13) and (14):

(13) $$c_{1}=2\times erf\left(0.5-\frac{t}{T}\right)$$

(14) $$c_{2}=3\times erf\left(1.7-\frac{t}{T}\right)$$

where *t* is the current iteration number and *T* is the maximum number of iterations.

**Figure 1:**
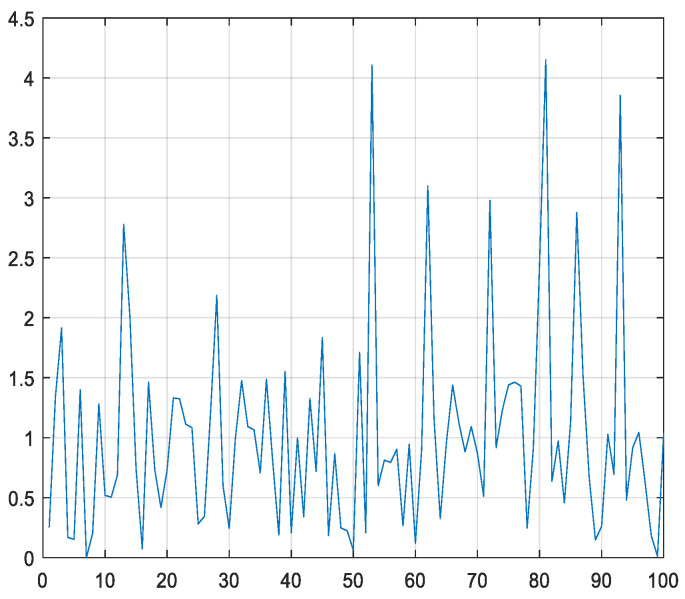
The iteration curve of α.

**Figure 2:**
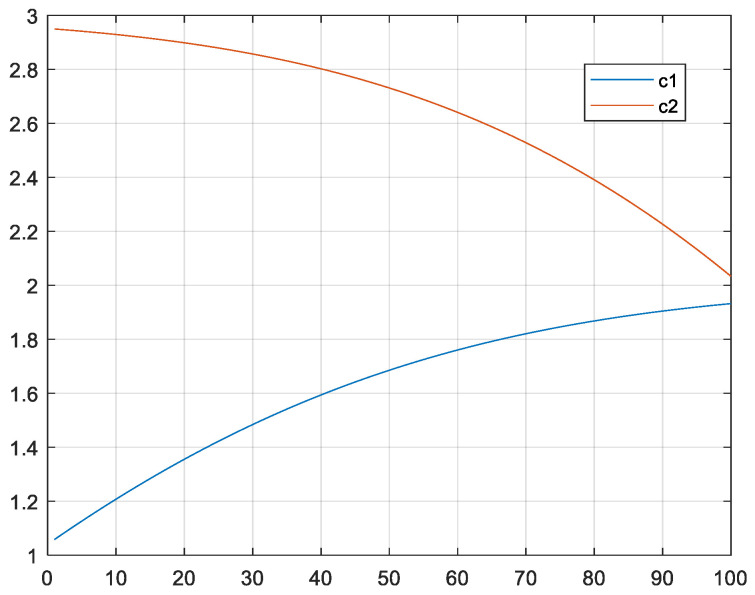
Iteration curves of *c*_1_ and *c*_2_.

The MRFO algorithm uses the parameter *α* to adjust the learning rate of individuals during the chain foraging phase, with the *α* value in each iteration shown in Figure 1. As can be seen from the figure, the range of *α* values extends beyond that of the interval [0, 1], and there is a high probability that it will be greater than 1. Therefore, at this stage, each individual tends to learn from the globally optimal individual, indicating that the algorithm is inclined towards global exploration. To enable particles in BPSO to learn in a similar manner, this section adjusts the learning factors *c*_1_ and *c*_2_, with their iteration curve diagrams shown in Figure 2. As can be seen from the figure, the value of *c*_2_ is greater than that of *c*_1_, which is beneficial for the algorithm’s exploitation. Both of them will change with the number of iterations. With the increasing number of iterations, the algorithm’s exploitation ability gradually weakens while its exploration ability gradually strengthens.

Then, we use a V-shaped transfer function to convert the particle’s position to the binary search space, as shown in Equations (15) and (16):

(15) $$Tf\left(v_{i}^{t+1}\right)=\left|\tanh\left(v_{i}^{t+1}\right)\right|$$

(16) $$x_{i}^{t+1}=\left\{\begin{array}{c} {\sim x}_{i}^{t}\;\;\mathrm{If}\;rand<\mathrm{Tf}\\ x_{i}^{t}\;\;\;\mathrm{If}\;rand\geq \mathrm{Tf} \end{array}\right.$$

where $x_{i}^{t}$ is the position of the *i*-th particle in the previous iteration.

### 3.2. Cyclone Learning

Cyclone learning includes two parts. In the first part, all particles except for the first one learn not only from the previous particle but also from the best particle. The velocity update formula is shown in Equation (17):

(17) $$v_{i}^{t+1}=\left\{\begin{array}{c} \omega v_{i}^{t}+c_{3}{\times r}_{1}\times \left({pbest}_{i}-x_{i}^{t}\right)+c_{4}{\times r}_{2}\times \left(gbest-x_{i}^{t}\right)\;\;\;i=1\\ \omega v_{i}^{t}+c_{3}{\times r}_{1}\times \left({pbest}_{i-1}-x_{i}^{t}\right)+c_{4}{\times r}_{2}\times \left(gbest-x_{i}^{t}\right)\;\;\;i=2,\ldots ,N \end{array}\right.$$

where *c*_3_ and *c*_4_ are learning factors. The updated formulas of *c*_3_ and *c*_4_ are shown in Equations (18) and (19):

(18) $$c_{3}=3\times erf\left(1.7-\frac{t}{T}\right)$$

(19) $$c_{4}=2\times erf\left(0.5-\frac{t}{T}\right)$$

From Equations (18) and (19), it is shown that *c*_3_ has a higher value than *c*_4_, which is a benefit for the algorithm’s exploration. These two values will change with an increasing number of iterations. The algorithm’s exploration ability gradually diminishes with increasing iterations, while its exploitation ability gradually strengthens.

In the second part, to facilitate a global search, we assign a new random position in the search space as the reference position for each particle, which forces them to seek a new position far from the current best one. Its mathematical equation is shown in Equations (20) and (21):

(20) $$x_{r}=LW+rand\times \left(UP-Lw\right)$$

(21) $$v_{i}^{t+1}=\left\{\begin{array}{c} \omega v_{i}^{t}+c_{5}{\times r}_{1}\times \left({pbest}_{i}-x_{i}^{t}\right)+c_{6}{\times r}_{2}\times \left(x_{r}-x_{i}^{t}\right)\;\;\;i=1\\ \omega v_{i}^{t}+c_{5}{\times r}_{1}\times \left({pbest}_{i-1}-x_{i}^{t}\right)+c_{6}{\times r}_{2}\times \left(x_{r}-x_{i}^{t}\right)\;\;\;\;\;i=2,\ldots ,N \end{array}\right.$$

where *x_r_* is a random position generated in the search space; *LW* and *UP* are the lower and upper limits of the search space, respectively; *c*_5_ and *c*_6_ are constants, and their values are both 2; and rand is a random number in the interval [0, 1].

### 3.3. Somersault Learning

In the somersault learning phase, the global best position is viewed as a pivot, and particles roll back and forth near the pivot and their personal best position. The position update formula for this phase can be expressed as Equations (22) and (23):

(22) $$x_{i}^{new}={pbest}_{i}^{t}+2\times \left(r_{1}\times gbest-r_{2}\times {pbest}_{i}^{t}\right)$$

(23) $$x_{i}^{t+1}=\left\{\begin{array}{c} 1{\;\;\;\;x}_{i}^{new}>0.5\\ 0\;\;\;\;x_{i}^{new}<0.5 \end{array}\right.$$

where $x_{i}^{new}$ is the new position of the *i*-th particle generated after somersault foraging learning, and its value is a continuous value. Then, it is converted into a binary value by comparing it with the threshold 0.5.

Equation (22) allows each particle to move to a new position between its personal best position and the global best position found so far. As the distance between the two positions decreases, the perturbation on the current position also decreases, and all particles gradually become close to the optimal solution. This adaptive reduction as iterations increase enhances the algorithm’s exploration ability.

### 3.4. Description of the BPSO-MRFL Algorithm

**Figure 3:**
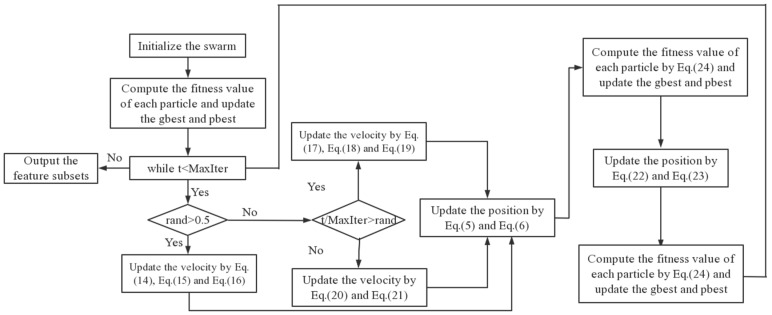
The flowchart of MRFL-BPSO.

The pseudocode of the BPSO-MRFL algorithm is presented in Algorithm 1. In Algorithm 1, lines 7~14 are the pseudocode of the cyclone foraging phase. Lines 15~18 are the pseudocode of the chain learning phase, and lines 22~24 are the pseudocode of the somersault learning phase. The flowchart of the BPSO-MRFL algorithm is shown in Figure 3.
**Algorithm 1:** Pseudocode of the BPSO-MRFL-based FS method.
**Input:** Maximum number of generations *T*, swarm size *N*, the dataset with *D* features;
**Output:** A set of selected features;1Initialize each particle’s position and *t* = 1;2Evaluate the fitness value of each particle using Equation (24);3Update the pbests and gbest;4**while** *t* < *T* **do**5**  for** *i* = 1 to *N* **do**6**    if** *rand* < 0.5 **then**7**      if** *t*/*T* < *rand* **then**//Cyclone foraging8$\;\;\;\;\;\;\;\;\mathrm{Update}\;x_{r}$ by Equation (20);9$\;\;\;\;\;\;\;\;\mathrm{Update}\;v_{i}^{t+1}$ by Equation (21);10$\;\;\;\;\;\;\;\;\mathrm{Update}\;x_{i}^{t+1}$ by Equations (15) and (16);11**      else**12$\;\;\;\;\;\;\;\;\mathrm{Update}\;v_{i}^{t+1}$ by Equations (17)–(19);13$\;\;\;\;\;\;\;\;\mathrm{Update}\;x_{i}^{t+1}$ by Equations (15) and (16);14**      end**15**    else**//Chain learning16$\;\;\;\;\;\;\mathrm{Update}\;v_{i}^{t+1}$ by Equations (12)–(14);17$\;\;\;\;\;\;\mathrm{Update}\;x_{i}^{t+1}$ by Equations (15) and (16);18**    end**19**  end**20**  **Evaluate the fitness value of each particle using Equation (24);21**  **Update the pbests and gbest;22**  for** *i* = 1 to *N* **do**//Somersault learning23$\;\;\;\;\mathrm{Update}\;x_{i}^{t+1}$ by Equations (22) and (23);24**  end**25**  **Evaluate the fitness value of each particle using Equation (24);26**  **Update the pbests and gbest;27**end**28**return gbest;**

## 4. Experiment Design

### 4.1. Datasets

This study used 10 gene expression datasets to rigorously evaluate the effectiveness of our proposed algorithm for feature selection [40]. A brief description and statistics about the datasets are shown in Table 1, including the number of features, instances of samples, and classes. These datasets are commonly used in research on feature selection due to their high dimensionality and small sample size. They have been extensively studied and applied in various research works.

### 4.2. Evaluating the Fitness

The purpose of feature selection is to enhance classification accuracy and minimize the number of selected features, which constitutes a multi-objective optimization problem. To address these objectives, a fitness function is devised using the linear weighting method, which is shown in Equation (24):

(24) $$fitness=\omega \times E+\left(1-\omega \right)\times \frac{d}{D}$$

where *E* is the classification error rate of a certain classifier, and *d* and *D* represent the number of selected features and the total number of features, respectively. Additionally, ω is a constant and its value is usually set to 0.99.

The wrapper method is implemented in this study using the k-nearest neighbor (KNN) algorithm to evaluate the classification accuracy. The KNN algorithm is an instance-based machine learning algorithm that classifies a new sample into the class of the k-nearest training data points based on their distance. KNN is a non-parametric algorithm since it does not require assumptions or modeling of the data beforehand. In this paper, each dataset was randomly divided into two parts: the training set accounted for 80%, and the remaining 20% was used as the test set. The value of k for the KNN algorithm was set to 5. The details of KNN can be learned in [41].

### 4.3. Parameter Settings

All the algorithms were executed with the same configurations on different datasets. The BPSO-MRFL algorithm was used for all experiments with the following settings: *T* = 100 for the maximum number of consecutive iterations, *N* = 20 for the population size, and a range of 0.4 to 0.9 for the inertia weight parameter.

## 5. Experiments and Discussion

In this section, we comprehensively evaluate and analyze the proposed BPSO-MRFL by experiments. We compare the BPSO-MRFL algorithm with several feature selection methods reported in the literature, including three classical filtering feature selection algorithms, four non-PSO-based feature selection methods, and four PSO variants reported in the recent literature. The experimental analysis and comparison are carried out in the following three aspects: classification accuracy, number of features, and fitness value. We use ten-fold cross-validation to construct training and test sets for our experiments. Specifically, one fold is reserved as unseen test data and is not used during the FS process. The remaining nine folds constitute the training data, which are exclusively used for FS. We evaluate the performance of the FS method through KNN classification. In these experiments, the parameters used for each method being compared were adjusted according to the values specified in the corresponding papers. And, we run all algorithm 20 times to analyze the results.

### 5.1. Comparative Analysis

#### 5.1.1. Comparison with Classical Feature Selection Methods

We conduct experiments to compare the performance of BPSO-MRFL with three traditional feature selection methods, namely CFS [42], FCBF [43], and LFS [44]. Table 2, Table 3 and Table 4 show the results of the experiments on ten different datasets. These results are the average value from the algorithm over 20 runs.

After analyzing and comparing the results presented in Table 1, it can be concluded that BPSO-MRFL is the best feature selection method for improving the performance of the classifier. On all 10 datasets, BPSO-MRFL achieves a higher classification accuracy than the other 3 classical feature selection methods. On 40% of the datasets, BPSO-MRFL outperforms the maximum classification accuracy obtained by the other methods by more than 5%. The datasets with the highest proportion of classification accuracy are 9Tumor and Brain Tumor2. These results provide strong evidence that BPSO-MRFL is superior to the other three classical feature selection methods in improving classifier performance.

Based on the data presented in Table 3, it can be concluded that the BPSO-MRFL algorithm is effective in eliminating redundant features. On the majority of the datasets, the optimal feature subset obtained by BPSO-MRFL has fewer features than other methods. On 60% of the datasets, BPSO-MRFL significantly reduces the number of features compared to other methods. Although the LFS algorithm obtains relatively smaller feature subsets on some datasets, the classification accuracy is lower. On the other hand, BPSO-MRFL achieves the best classification accuracy while obtaining a smaller feature subset, indicating that it is a robust method for most datasets. Therefore, the results fully demonstrate that BPSO-MRFL is the most effective method for eliminating redundant features compared to the three classical feature selection methods.

The experimental results presented in Table 4 demonstrate that the BPSO-MRFL algorithm outperforms other comparison algorithms on all 10 datasets, achieving the best average fitness values. This indicates that the proposed algorithm exhibits superior performance compared to the other methods.

#### 5.1.2. Comparison with Other Well-Known Optimizers

In this section, we compare the performance of BPSO-MRFL with other non-PSO well-known optimizers for feature selection tasks.

Specifically, we compare it with GA [45], HLBDA [46], BGWO2 [24], and BGSA [47] optimizers, and the results are presented in Table 5, Table 6, Table 7 and Table 8. These results are the average value and variance of the algorithm over 20 runs.

Upon comparing the data in Table 5, the results indicate significant improvements in the performance of the classifier. On the 10 datasets, BPSO-MRFL outperforms the other optimization algorithms in terms of classification accuracy on 7 datasets. Specifically, BPSO-MRFL demonstrates significantly higher classification accuracy than the other comparative algorithms on the Prostate Tumor and Brain Tumor2 datasets. On Leukemia1, DLBCL, and Leukemia3, the proposed algorithm and BGWO2 show the same average classification accuracy, but the average feature selection ratio of the proposed algorithm is significantly less than that of BGWO2. These results clearly indicate that BPSO-MRFL is the most effective among the five feature selection methods in enhancing the classifier’s performance.

The results of the elimination of redundant features are shown in Table 6. On all datasets, BPSO-MRFL obtains an optimal feature subset with a number of features that is less than 50% of the minimum value achieved by other methods. Furthermore, on 80% of the datasets, the number of features in the optimal subset obtained by BPSO-MRFL is below 20% of the minimum value obtained by other methods. On 30% of the datasets, this number is even below 10% of the minimum value obtained by other methods. And, BPSO-MRFL obtains the fewest features on Brain Tumor2. These findings provide strong evidence that the BPSO-MRFL method outperforms the other four optimizers in eliminating redundant features.

Based on the data presented in Table 7 and Table 8, it is evident that the BPSO-MRFL method achieves superior fitness values compared to the other four methods. Therefore, we can draw the following conclusion: in comparison to these non-PSO-based methods, the BPSO-MRFL algorithm proposed in this study has significant competitive advantages. That also substantiates the progressiveness of the BPSO-MRFL algorithm in dealing with the feature selection problem on high-dimensional data.

#### 5.1.3. Comparison with Different Variants of PSO

In this part, we compare BPSO-MRFL with some different variants of PSO: SBPSO [17], VBPSO [17], QBPSO [48], and UTF-BPSO [49]. The performances of BPSO-MRFL and other PSO-based methods are compared in Table 9, Table 10, Table 11 and Table 12. By comparing the results with those of SBPSO, VBPSO, and QBPSO, it is evident that BPSO-MRFL outperforms these algorithms on all datasets. BPSO-MRFL achieves significantly higher classification accuracy while reducing the number of features on three datasets, namely Leukemia1, DLBCL, and Leukemia3. These findings demonstrate that BPSO-MRFL is a more effective method for feature selection than the other three PSO-based algorithms.

When compared with UTF-BPSO, BPSO-MRFL achieves better FS results on all datasets. Although UTF-BPSO selected fewer features than SBPSO, VBPSO, and QBPSO, the optimal feature subset obtained by BPSO-MRFL has several times fewer features than UTF-BPSO, particularly on the Brain Tumor2 dataset, where the difference is 42.3 times. Moreover, the fitness values of BPSO-MRFL are higher than those of the other BPSO variants on all datasets.

#### 5.1.4. Overall Comparison on the Statistical Results and Radar Chart

In Table 13, the results of the Friedman test [50], a non-parametric statistical test, are present based on the classification accuracy, number of selected features, and fitness value obtained by the algorithms. The minimum value in the table indicates a better result. The algorithms are ranked in the test, and as seen in the table, BPSO-MRFL achieves the best rank. The Wilcoxon signed-rank test, as described in [51], is employed to perform pairwise comparisons between the methods. A *p*-value greater than 0.05 indicates similar classification performances between the two methods, while a *p*-value below 0.05 signifies significant differences. Table 14 presents the results of the Wilcoxon test conducted to evaluate BPSO-MRFL against other competing methods. The obtained results demonstrate that, in the majority of cases, the proposed BPSO-MRFL exhibits significantly superior classification performance compare to the other methods. These results demonstrate that the introduced learning strategies significantly enhance the efficiency of BPSO-MRFL in binary search spaces.

### 5.2. Convergence Analysis

**Figure 4:**
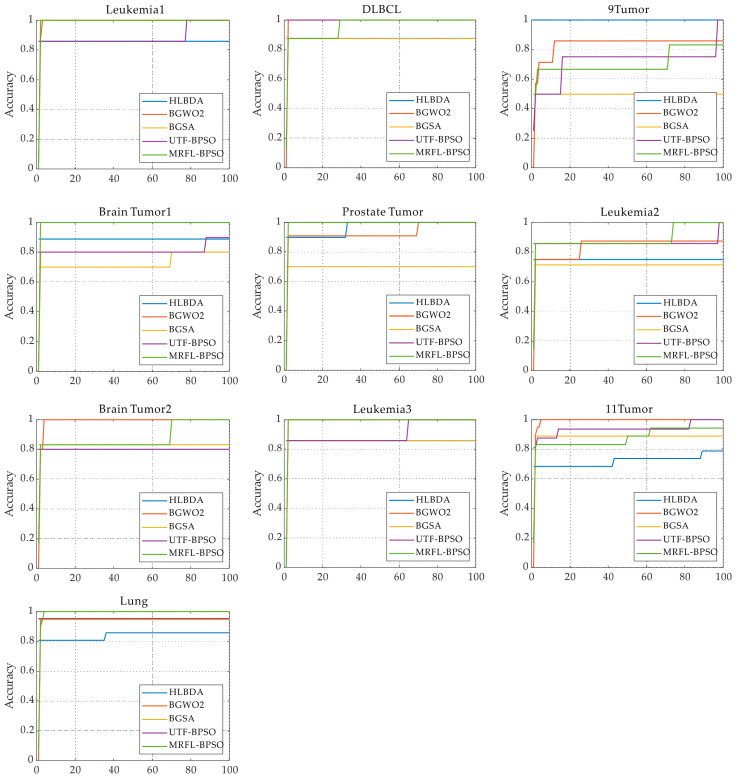
Convergence curves of the MRFL-BPSO algorithms and other comparison algorithms on each dataset.

Figure 4 shows the convergence curves of BPSO-MRFL and several comparison algorithms. Each curve represents the result of 1 iteration among 20 runs. We plot the convergence curves of HLBDA, BGWO2, BGSA and UTF-BPSO and compare them with BPSO-MRFL. We chose HLBDA, BGWO2, BGSA, and UTF-BPSO because these algorithms have an average rank in the top five of the Friedman experiment. From Figure 4, it can be seen that the convergence curve of BPSO-MRFL significantly outperforms the other algorithms on most datasets, indicating that BPSO-MRFL has a better convergence ability. On the Leukemia1, Brain Tumor1, Prostate Tumor, Leukemia3, and Lung datasets, BPSO-MRFL has a fast convergence speed and achieves higher or comparable accuracy than the other comparison algorithms. On the DLBCL, Leukemia2, and Brain Tumor2 datasets, the convergence speed of the BPSO-MRFL algorithm in the early iterations is slower than that of other comparison algorithms, but it achieves the same classification accuracy as other the comparison algorithms in the later iterations. As shown in Table 6 and Table 10, the number of features selected by BPSO-MRFL on these three datasets is much lower than that of the other comparison algorithms. This all indicates that the chain learning and cyclone learning strategies enhance the algorithm’s exploration and exploitation abilities. In addition, we can also observe from Figure 4 that the somersault learning strategy allows BPSO-MRFL to jump out of local optima in the later iterations.

**Figure 5:**
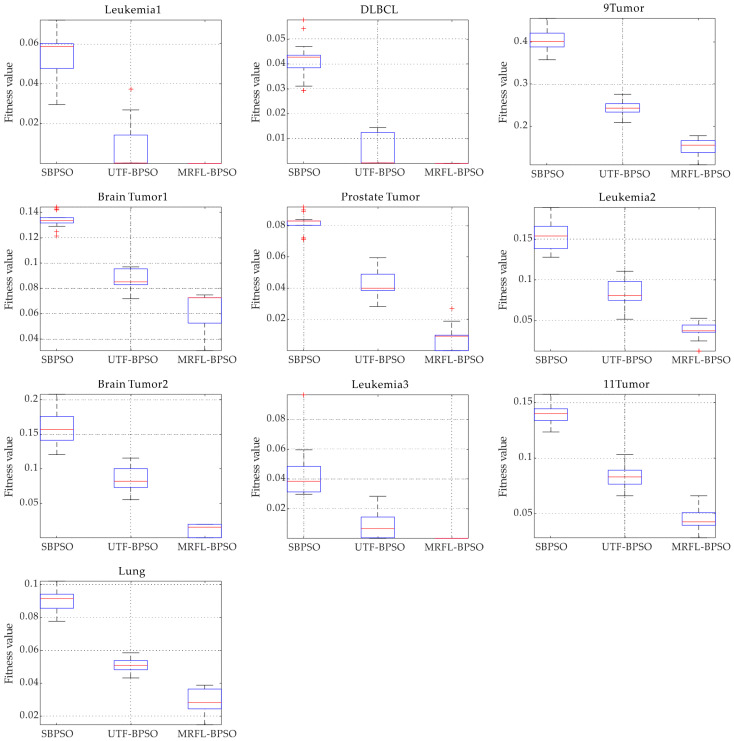
Boxplots of fitness values for MRFL-BPSO versus SBPSO and UTF-BPSO in dealing with the 10 datasets.

On the other hand, Figure 5 presents the boxplot of SBPSO, UTF-BPSO, and BPSO-MRFL. From Figure 5, it can be observed that BPSO-MRFL outperforms SBPSO and UTF-BPSO in terms of both median and mean values. These results strongly support the effectiveness of the proposed learning strategy in achieving the highest prediction accuracy in this study. Moreover, the boxplots also indicate that the proposed algorithm exhibits high stability on most of the datasets, providing further evidence of its superior convergence ability compared to other algorithms.

**Figure 6:**
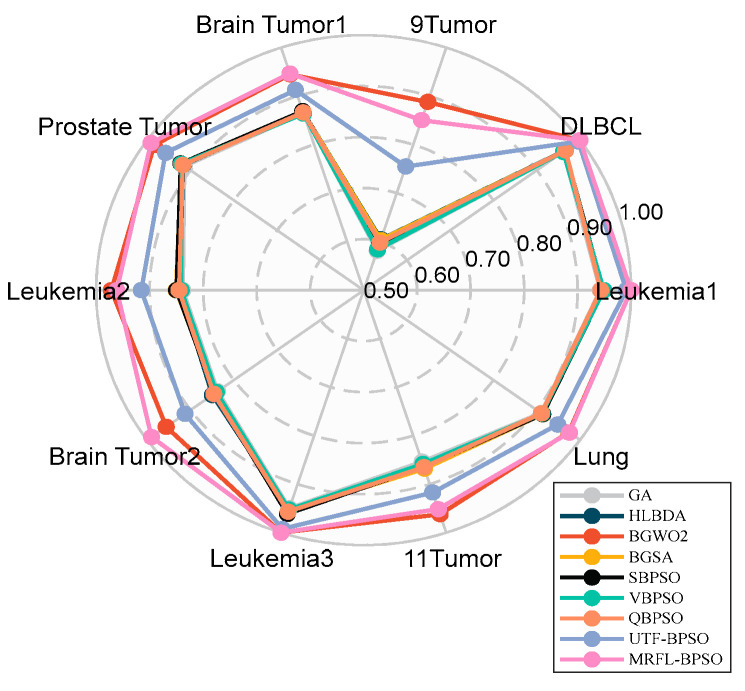
Comparison of MRFL-BPSO against other methods in terms of average accuracy.

**Figure 7:**
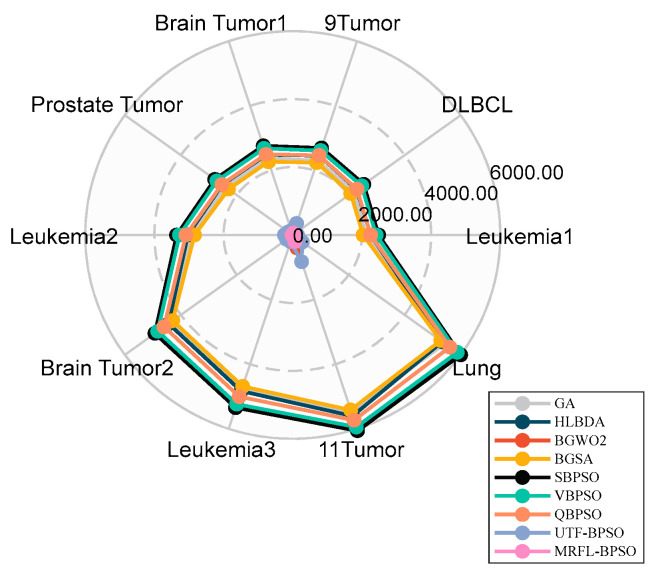
Comparison of MRFL-BPSO against other methods in terms of average feature subset size.

Figure 6 presents the average classification accuracy of MRFL-BPSO compared with other algorithms for the different datasets. As illustrated in Figure 6, MRFL-BPSO achieves superior classification accuracy over the other algorithms on the datasets except 9Tumor. For the 9Tumor dataset, while MRFL-BPSO is outperformed by BGWO2, it still maintains higher accuracy than the remaining algorithms. Figure 7 demonstrates the size of selected feature subsets obtained by MRFL-BPSO in comparison with other algorithms. The results reveal that MRFL-BPSO selects significantly smaller feature subsets than six algorithms (VPSO, SBPSO, QBPSO, BGSA, UTF-BPSO, and HLBDA), while yielding comparable subset sizes to the other two algorithms (GA and BGWO2). Therefore, these findings provide full evidence for the effectiveness of the MRFL-BPSO algorithm.

Based on the above comparison, it can be concluded that the three proposed learning strategies are effective and BPSO-MRFL has competitive performance in terms of convergence.

## 6. Conclusions

In this paper, a new algorithm called BPSO-MRFL has been proposed. The algorithm is based on the manta ray foraging search mechanism and has three different search phases: chain learning, cyclone learning, and somersault learning. The chain learning phase enhances the social learning ability of the population. In the cyclone learning phase, the gradual increase in iterations encourages BPSO-MRFL to smoothly transition from an exploratory search to an exploitative search, balancing the algorithm’s exploration and exploitation abilities. The somersault learning phase allows particles to adaptively search in a changing search range, enhancing the local search ability of the algorithm. Therefore, all three search phases contribute to improving the search performance of BPSO-MRFL.

The proposed BPSO-MRFL algorithm has been comprehensively evaluated on gene expression datasets and compared with well-established swarm optimization algorithms, including three classical feature selection, four well-known optimizers, and four different variants of PSO. The experimental results demonstrate that BPSO-MRFL can achieve competitive or even better performance on most datasets. The algorithm combined with the classifier studied in this paper is only the KNN classifier with a few parameters and easy to implement. There are many classifiers that affect the classification performance of feature selection. How to select the optimal classifier needs further research. In future work, we will aim to conduct a deeper study of the BPSO-MRFL algorithm’s performance on ultra-high dimensional datasets and further improve its performance and enhance its efficiency.

## Figures and Tables

**Table 1:** Summary of the experimental datasets.

ID	Datasets	Instances	Features	Classes
1	Leukemia1	72	5327	3
2	DLBCL	77	5469	2
3	9Tumor	60	5726	9
4	Brain Tumor1	90	5920	5
5	Prostate Tumor	102	10,509	2
6	Leukemia2	72	7129	3
7	Brain Tumor2	50	10,367	4
8	Leukemia3	72	11,225	3
9	11Tumor	174	12,533	11
10	Lung	203	12,600	5

**Table 2:** Comparison of classification accuracy with classical feature selection methods.

Datasets	CFS	FCBF	LFS	MRFL-BPSO
Leukemia1	0.9694	0.9516	0.9766	**1.0000**
DLBCL	0.9921	0.9843	0.9708	**1.0000**
9Tumor	0.6377	0.6230	0.5726	**0.8504**
Brain Tumor1	0.9148	0.9129	0.8841	**0.9467**
Prostate Tumor	0.9547	0.9511	0.9435	**0.9914**
Leukemia2	0.9256	0.9350	0.9247	**0.9616**
Brain Tumor2	0.8515	0.8764	0.8113	**0.9900**
Leukemia3	0.9979	0.9967	0.9468	**1.0000**
11Tumor	0.9024	0.9062	0.8260	**0.9516**
Lung	0.9677	0.9661	0.9190	**0.9754**

Note: Bold indicates the best results.

**Table 3:** Comparison of the number of features with classical feature selection methods.

Datasets	CFS	FCBF	LFS	MRFL-BPSO
Leukemia1	97	49	22	**7.96**
DLBCL	88	66	18	**3.14**
9Tumor	47	32	**26**	57.76
Brain Tumor1	142	106	22	**7.45**
Prostate Tumor	59	49	13	**6.80**
Leukemia2	119	71	21	**17.19**
Brain Tumor2	117	75	**5**	5.10
Leukemia3	138	80	15	**5.13**
11Tumor	379	394	**25**	200.53
Lung	550	453	**10**	20.31

Note: Bold indicates the best results.

**Table 4:** Comparison of fitness values with classical feature selection methods.

Datasets	CFS	FCBF	LFS	MRFL-BPSO
Leukemia1	0.03047	0.04800	0.02319	**0.00002**
DLBCL	0.00794	0.01571	0.02894	**0.00001**
9Tumor	0.35875	0.37330	0.42320	**0.14448**
Brain Tumor1	0.08454	0.08638	0.11474	**0.05365**
Prostate Tumor	0.04496	0.04850	0.05591	**0.00949**
Leukemia2	0.07382	0.06450	0.07456	**0.03894**
Brain Tumor2	0.14718	0.12242	0.18682	**0.01074**
Leukemia3	0.00219	0.00334	0.05264	**0.00001**
11Tumor	0.09695	0.09316	0.17228	**0.04822**
Lung	0.03240	0.03390	0.08016	**0.02436**

Note: Bold indicates the best results.

**Table 5:** Comparison of classification accuracy with other well-known optimizers.

Datasets	GA	HLBDA	BGWO2	BGSA	MRFL-BPSO
Leukemia1	0.9424 ± 0.01	0.9477 ± 0.01	**1.0000 ± 0.00**	0.9475 ± 0.01	**1.0000 ± 0.00**
DLBCL	0.9619 ± 0.01	0.9656 ± 0.01	**1.0000 ± 0.00**	0.9644 ± 0.01	**1.0000 ± 0.00**
9Tumor	0.5813 ± 0.03	0.6047 ± 0.02	**0.8881 ± 0.02**	0.6034 ± 0.04	0.8504 ± 0.02
Brain Tumor1	0.8650 ± 0.01	0.8677 ± 0.01	0.9448 ± 0.01	0.8685 ± 0.01	**0.9467 ± 0.01**
Prostate Tumor	0.9142 ± 0.01	0.9174 ± 0.01	0.9838 ± 0.01	0.9215 ± 0.01	**0.9914 ± 0.01**
Leukemia2	0.8381 ± 0.01	0.8442 ± 0.01	**0.9716 ± 0.01**	0.8508 ± 0.02	0.9914 ± 0.01
Brain Tumor2	0.8375 ± 0.02	0.8495 ± 0.02	0.9563 ± 0.02	0.8433 ± 0.03	**0.9900 ± 0.01**
Leukemia3	0.9496 ± 0.01	0.9538 ± 0.01	**1.0000 ± 0.00**	0.9553 ± 0.02	**1.0000 ± 0.00**
11Tumor	0.8536 ± 0.01	0.8618 ± 0.01	**0.9622 ± 0.01**	0.8681 ± 0.01	0.9516 ± 0.01
Lung	0.9105 ± 0.00	0.9114 ± 0.00	0.9742 ± 0.01	0.9138 ± 0.00	**0.9754 ± 0.01**

Note: Bold indicates the best results.

**Table 6:** Comparison of the number of features with other well-known optimizers.

Datasets	GA	HLBDA	BGWO2	BGSA	MRFL-BPSO
Leukemia1	2114.68 ± 7.17	2253.52 ± 25.08	54.07 ± 5.26	2037.37 ± 10.83	**7.96 ± 3.06**
DLBCL	2165.74 ± 8.40	2281.01 ± 26.62	36.54 ± 3.59	2076.37 ± 8.40	**3.14 ± 0.66**
9Tumor	2304.79 ± 11.19	2456.16 ± 36.92	146.44 ± 13.83	2241.27 ± 23.82	**57.76 ± 19.06**
Brain Tumor1	2366.83 ± 11.16	2481.96 ± 30.69	60.92 ± 6.19	2271.22 ± 11.68	**7.45 ± 4.10**
Prostate Tumor	2393.32 ± 7.88	2516.43 ± 31.38	66.97 ± 7.62	2297.80 ± 13.20	**6.80 ± 3.17**
Leukemia2	2926.30 ± 8.05	3030.57 ± 36.19	102.74 ± 8.68	2841.34 ± 15.43	**17.19 ± 7.68**
Brain Tumor2	4405.86 ± 11.78	4441.26 ± 46.54	100.90 ± 12.23	4289.51 ± 13.66	**5.10 ± 1.39**
Leukemia3	4805.86 ± 13.78	4832.15 ± 51.71	83.26 ± 8.70	4688.20 ± 22.83	**5.13 ± 0.97**
11Tumor	5470.28 ± 21.20	5605.70 ± 58.50	400.94 ± 23.75	5415.72 ± 26.71	**200.53 ± 36.36**
Lung	5427.71 ± 9.53	5345.34 ± 66.72	167.43 ± 21.27	5288.86 ± 18.85	**20.31 ± 5.27**

Note: Bold indicates the best results.

**Table 7:** Comparison of fitness values with other well-known optimizers.

Datasets	GA	HLBDA	BGWO2	BGSA	MRFL-BPSO
Leukemia1	0.06104 ± 0.01	0.05597 ± 0.01	0.00010 ± 0.00	0.05585 ± 0.01	**0.00002 ± 0.00**
DLBCL	0.04164 ± 0.01	0.03820 ± 0.01	0.00007 ± 0.00	0.03907 ± 0.01	**0.00001 ± 0.00**
9Tumor	0.41856 ± 0.03	0.39567 ± 0.02	**0.11104 ± 0.02**	0.39652 ± 0.04	0.14448 ± 0.02
Brain Tumor1	0.13766 ± 0.01	0.13522 ± 0.01	0.05472 ± 0.01	0.13406 ± 0.01	**0.05365 ± 0.01**
Prostate Tumor	0.08897 ± 0.01	0.08603 ± 0.01	0.01613 ± 0.01	0.08161 ± 0.01	**0.00949 ± 0.01**
Leukemia2	0.16439 ± 0.01	0.15852 ± 0.01	**0.02826 ± 0.01**	0.15166 ± 0.02	0.03894 ± 0.01
Brain Tumor2	0.16510 ± 0.02	0.15330 ± 0.02	0.04332 ± 0.02	0.15925 ± 0.03	**0.01074 ± 0.01**
Leukemia3	0.05422 ± 0.01	0.05006 ± 0.01	0.00007 ± 0.00	0.04846 ± 0.02	**0.00001 ± 0.00**
11Tumor	0.14926 ± 0.01	0.14131 ± 0.01	**0.03770 ± 0.01**	0.13487 ± 0.01	0.04822 ± 0.01
Lung	0.09291 ± 0.00	0.09195 ± 0.00	0.02565 ± 0.01	0.08957 ± 0.00	**0.02436 ± 0.01**

Bold indicates the best results.

**Table 8:** Comparison of the statistical variance of fitness values with other well-known optimizers.

Datasets	GA	HLBDA	BGWO2	BGSA	MRFL-BPSO
Leukemia1	8.14 × 10^−3^	1.01 × 10^−2^	9.88 × 10^−6^	9.99 × 10^−3^	**8.29 × 10^−6^**
DLBCL	8.53 × 10^−3^	1.00 × 10^−2^	6.57 × 10^−6^	1.09 × 10^−2^	**1.45 × 10^−6^**
9Tumor	2.77 × 10^−2^	1.95 × 10^−2^	**1.71 × 10^−2^**	3.88 × 10^−2^	2.06 × 10^−2^
Brain Tumor1	1.09 × 10^−2^	**5.79 × 10^−3^**	1.12 × 10^−2^	8.42 × 10^−3^	1.35 × 10^−2^
Prostate Tumor	9.84 × 10^−3^	**6.13 × 10^−3^**	8.54 × 10^−3^	6.78 × 10^−3^	8.32 × 10^−3^
Leukemia2	1.35 × 10^−2^	1.47 × 10^−2^	**8.20 × 10^−3^**	1.74 × 10^−2^	1.24 × 10^−2^
Brain Tumor2	2.33 × 10^−2^	2.39 × 10^−2^	1.58 × 10^−2^	2.61 × 10^−2^	**9.65 × 10^−3^**
Leukemia3	1.41 × 10^−2^	1.39 × 10^−2^	7.75 × 10^−6^	1.56 × 10^−2^	**9.76 × 10^−7^**
11Tumor	1.31 × 10^−2^	1.16 × 10^−2^	**1.06 × 10^−2^**	1.19 × 10^−2^	1.11 × 10^−2^
Lung	4.91 × 10^−3^	**4.32 × 10^−3^**	5.90 × 10^−3^	4.32 × 10^−3^	7.20 × 10^−3^

Bold indicates the best results.

**Table 9:** Comparison of classification accuracy with different variants of PSO.

Datasets	SBPSO	VBPSO	QBPSO	UTF-BPSO	MRFL-BPSO
Leukemia1	0.9484 ± 0.01	0.9491 ± 0.01	0.9433 ± 0.01	0.9911 ± 0.01	**1.0000 ± 0.00**
DLBCL	0.9629 ± 0.01	0.9615 ± 0.01	0.9663 ± 0.01	0.9961 ± 0.01	**1.0000 ± 0.00**
9Tumor	0.5964 ± 0.02	0.5841 ± 0.03	0.5983 ± 0.03	0.7548 ± 0.02	**0.8504 ± 0.02**
Brain Tumor1	0.8691 ± 0.01	0.8638 ± 0.01	0.8664 ± 0.01	0.9128 ± 0.01	**0.9467 ± 0.01**
Prostate Tumor	0.9225 ± 0.01	0.9210 ± 0.00	0.9180 ± 0.01	0.9578 ± 0.01	**0.9914 ± 0.01**
Leukemia2	0.8497 ± 0.02	0.8410 ± 0.02	0.8453 ± 0.01	0.9158 ± 0.02	**0.9914 ± 0.01**
Brain Tumor2	0.8448 ± 0.02	0.8402 ± 0.02	0.8462 ± 0.03	0.9128 ± 0.02	**0.9900 ± 0.01**
Leukemia3	0.9612 ± 0.02	0.9524 ± 0.02	0.9567 ± 0.02	0.9924 ± 0.01	**1.0000 ± 0.00**
11Tumor	0.8641 ± 0.01	0.8583 ± 0.01	0.8650 ± 0.01	0.9171 ± 0.01	**0.9516 ± 0.01**
Lung	0.9137 ± 0.01	0.9118 ± 0.01	0.9107 ± 0.00	0.9484 ± 0.00	**0.9754 ± 0.01**

Note: Bold indicates the best results.

**Table 10:** Comparison of the number of features with different variants of PSO.

Datasets	SBPSO	VBPSO	QBPSO	UTF-BPSO	MRFL-BPSO
Leukemia1	2482.87 ± 7.28	2410.29 ± 10.04	2253.32 ± 9.53	138.45 ± 24.35	**7.96 ± 3.06**
DLBCL	2538.26 ± 7.24	2463.62 ± 9.54	2296.30 ± 9.42	97.83 ± 13.42	**3.14 ± 0.66**
9Tumor	2694.09 ± 11.4	2613.56 ± 8.64	2463.75 ± 15.34	362.47 ± 65.11	**57.76 ± 19.06**
Brain Tumor1	2760.83 ± 8.01	2679.51 ± 12.46	2498.81 ± 10.76	135.60 ± 26.36	**7.45 ± 4.10**
Prostate Tumor	2786.19 ± 9.84	2711.89 ± 15.03	2531.60 ± 9.77	156.56 ± 30.77	**6.80 ± 3.17**
Leukemia2	3357.62 ± 15.29	3272.85 ± 12.98	3088.87 ± 17.92	240.89 ± 32.49	**17.19 ± 7.68**
Brain Tumor2	4926.52 ± 13.57	4823.65 ± 13.28	4600.55 ± 15.64	215.85 ± 24.67	**5.10 ± 1.39**
Leukemia3	5346.87 ± 12.62	5237.68 ± 17.4	5003.23 ± 14.94	208.33 ± 23.98	**5.13 ± 0.97**
11Tumor	6059.46 ± 16.99	5947.66 ± 19.14	5731.80 ± 21.88	828.62 ± 144.87	**200.53 ± 36.36**
Lung	6002.85 ± 11.37	5877.43 ± 8.84	5616.36 ± 14.22	344.80 ± 81.67	**20.31 ± 5.27**

Note: Bold indicates the best results.

**Table 11:** Comparison of fitness values with different variants of PSO.

Datasets	SBPSO	VBPSO	QBPSO	UTF-BPSO	MRFL-BPSO
Leukemia1	0.05578 ± 0.01	0.05494 ± 0.01	0.06036 ± 0.01	0.00903 ± 0.01	**0.00002 ± 0.00**
DLBCL	0.04135 ± 0.01	0.04263 ± 0.01	0.03755 ± 0.01	0.00407 ± 0.01	**0.00001 ± 0.00**
9Tumor	0.40426 ± 0.02	0.41632 ± 0.03	0.40194 ± 0.03	0.24335 ± 0.02	**0.14448 ± 0.02**
Brain Tumor1	0.13423 ± 0.01	0.13932 ± 0.01	0.13647 ± 0.01	0.08652 ± 0.01	**0.05365 ± 0.01**
Prostate Tumor	0.08144 ± 0.01	0.08276 ± 0.00	0.08538 ± 0.01	0.04202 ± 0.01	**0.00949 ± 0.01**
Leukemia2	0.15347 ± 0.02	0.16200 ± 0.02	0.15750 ± 0.01	0.08374 ± 0.02	**0.03894 ± 0.01**
Brain Tumor2	0.15838 ± 0.02	0.16285 ± 0.02	0.15670 ± 0.03	0.08657 ± 0.02	**0.01074 ± 0.01**
Leukemia3	0.04321 ± 0.02	0.05181 ± 0.02	0.04736 ± 0.02	0.00773 ± 0.01	**0.00001 ± 0.00**
11Tumor	0.13942 ± 0.01	0.14498 ± 0.01	0.13826 ± 0.01	0.08275 ± 0.01	**0.04822 ± 0.01**
Lung	0.09016 ± 0.01	0.09202 ± 0.01	0.09286 ± 0.00	0.05134 ± 0.00	**0.02436 ± 0.01**

Note: Bold indicates the best results.

**Table 12:** Comparison of the statistical variance of fitness values with different variants of PSO.

Datasets	SBPSO	VBPSO	QBPSO	UTF-BPSO	MRFL-BPSO
Leukemia1	1.03 × 10^−2^	1.13 × 10^−2^	8.13 × 10^−3^	1.15 × 10^−2^	**8.29 × 10^−6^**
DLBCL	7.49 × 10^−3^	1.01 × 10^−2^	9.97 × 10^−3^	6.11 × 10^−3^	**1.45 × 10^−6^**
9Tumor	2.47 × 10^−2^	2.86 × 10^−2^	3.04 × 10^−2^	**1.91 × 10^−2^**	2.06 × 10^−2^
Brain Tumor1	**5.84 × 10^−3^**	8.23 × 10^−3^	9.18 × 10^−3^	7.33 × 10^−3^	1.35 × 10^−2^
Prostate Tumor	5.86 × 10^−3^	**4.00 × 10^−3^**	9.43 × 10^−3^	9.70 × 10^−3^	8.32 × 10^−3^
Leukemia2	1.66 × 10^−2^	1.57 × 10^−2^	**1.06 × 10^−2^**	1.60 × 10^−2^	1.24 × 10^−2^
Brain Tumor2	2.45 × 10^−2^	1.98 × 10^−2^	2.66 × 10^−2^	1.70 × 10^−2^	**9.65 × 10^−3^**
Leukemia3	1.61 × 10^−2^	1.88 × 10^−2^	1.67 × 10^−2^	8.42 × 10^−3^	**9.76 × 10^−7^**
11Tumor	**8.35 × 10^−3^**	1.17 × 10^−2^	1.05 × 10^−2^	9.52 × 10^−3^	1.11 × 10^−2^
Lung	6.29 × 10^−3^	5.67 × 10^−3^	4.67 × 10^−3^	**4.59 × 10^−3^**	7.20 × 10^−3^

Note: Bold indicates the best results.

**Table 13:** Overall rank by the F-test for all algorithm based on accuracy, number of features, and fitness value.

Algorithm	GA	HLBDA	BGWO2	BGSA	SBPSO	VBPSO	BQPSO	UTF-BPSO	MRFL-BPSO
Accuracy	8.8	6.1	1.55	5.3	5.3	7.4	6.1	3	**1.45**
Features	5.1	6	2	4	9	8	6.9	3	**1**
Fitness	8.8	6.1	1.7	5.1	5.4	7.5	6.1	3	**1.3**
Average rank	7.57	6.07	1.75	4.8	6.57	7.63	6.37	3	**1.25**
Finally rank	8	5	2	4	7	9	6	3	**1**

Note: Bold indicates the best results.

**Table 14:** The results of the Wilcoxon test of the proposed MRFL-BPSO against other methods.

Dataset	Algorithm							
	GA	HLBDA	BGWO2	BGSA	SBPSO	VBPSO	BQPSO	UTF-BPSO
Leukemia1	7.90 × 10^−9^	7.89 × 10^−9^	NaN	7.83 × 10^−9^	7.82 × 10^−9^	7.89 × 10^−9^	7.48 × 10^−9^	9.25 × 10^−4^
DLBCL	7.29 × 10^−9^	7.45 × 10^−9^	NaN	7.66 × 10^−9^	6.91 × 10^−9^	7.45 × 10^−9^	7.38 × 10^−9^	9.49 × 10^−3^
9Tumor	6.77 × 10^−8^	6.77 × 10^−8^	8.03 × 10^−6^	6.76 × 10^−8^	6.77 × 10^−8^	6.77 × 10^−8^	6.77 × 10^−8^	6.77 × 10^−8^
Brain Tumor1	6.54 × 10^−8^	6.57 × 10^−8^	7.75 × 10^−1^	6.55 × 10^−8^	6.53 × 10^−8^	6.56 × 10^−8^	6.46 × 10^−8^	1.24 × 10^−7^
Prostate Tumor	5.44 × 10^−8^	5.40 × 10^−8^	5.45 × 10^−3^	5.53 × 10^−8^	5.01 × 10^−8^	4.99 × 10^−8^	5.46 × 10^−8^	5.42 × 10^−8^
Leukemia2	6.73 × 10^−8^	6.73 × 10^−8^	6.78 × 10^−3^	6.74 × 10^−8^	6.73 × 10^−8^	6.73 × 10^−8^	6.71 × 10^−8^	1.89 × 10^−7^
Brain Tumor2	5.35 × 10^−8^	5.34 × 10^−8^	1.16 × 10^−6^	5.36 × 10^−8^	5.37 × 10^−8^	5.36 × 10^−8^	5.35 × 10^−8^	5.32 × 10^−8^
Leukemia3	7.70 × 10^−9^	7.88 × 10^−9^	NaN	7.85 × 10^−9^	7.68 × 10^−9^	7.90 × 10^−9^	7.68 × 10^−9^	3.93 × 10^−4^
11Tumor	6.79 × 10^−8^	6.79 × 10^−8^	8.35 × 10^−3^	6.79 × 10^−8^	6.79 × 10^−8^	6.79 × 10^−8^	6.79 × 10^−8^	1.23 × 10^−7^
Lung	6.76 × 10^−8^	6.75 × 10^−8^	4.40 × 10^−1^	6.76 × 10^−8^	6.75 × 10^−8^	6.75 × 10^−8^	6.75 × 10^−8^	6.75 × 10^−8^
Sum	10	10	5	10	10	10	10	10

## Data Availability

The data are contained within this article.

## Abbreviations

The following abbreviations are used in this manuscript:

BDA	Binary Dragonfly Algorithm
BOA	Butterfly Optimization Algorithm
BPSO	Binary Particle Swarm Optimization
BPSO-MRFL	Binary Particle Swarm Optimization with Manta Ray Foraging Learning Strategies
CFS	Correlation-based Feature Selection
CSO	Competitive Particle Swarm Optimization
FS	Feature Selection
GA	Genetic Algorithm
GAs	Genetic Algorithms
GOA	Grasshopper Optimization Algorithm
GSA	Gravitational Search Algorithm
GWO	Gray Wolf Optimization
MIBBPSO	Mutual Information-based Bare-Bones PSO algorithm
MRFO	Manta Ray Foraging Optimization
PSO	Particle Swarm Optimization
QBPSO	Quantum-Inspired Binary Particle Swarm Optimization
SBPSO	S-Shaped Transfer Function-based Binary Particle Swarm Optimization
UTF-BPSO	Upgrade Transfer Function for Binary Particle Swarm Optimization
VBPSO	V-Shaped Transfer Function-based Binary Particle Swarm Optimization
WOA	Whale Optimization Algorithm
